# Resistance Training of Inspiratory Muscles After Coronary Artery Disease May Improve Obstructive Sleep Apnea in Outpatient Cardiac Rehabilitation: RICAOS Study

**DOI:** 10.3389/fphys.2022.846532

**Published:** 2022-03-03

**Authors:** Pierre Labeix, Mathieu Berger, Amandine Zellag, Arnauld Garcin, Jean-Claude Barthelemy, Frederic Roche, David Hupin

**Affiliations:** ^1^SAINBIOSE, U1059 INSERM, University of Lyon, University Jean Monnet, Saint-Etienne, France; ^2^Department of Clinical and Exercise Physiology, University Hospital of Saint-Etienne, Saint-Etienne, France; ^3^Center for Investigation and Research in Sleep, CHUV and UNIL, Lausanne, Switzerland; ^4^Infectious Diseases Department, University Hospital of Saint-Etienne, Saint-Etienne, France; ^5^Innovation and Pharmacology Clinical Research Unit, University Hospital of Saint-Etienne, Saint-Etienne, France

**Keywords:** resistive inspiratory muscle training, obstructive sleep apnea, coronary artery disease, apnea-hypopnea index, cardiac rehabilitation, oxygen desaturation index

## Abstract

**Background:**

Obstructive sleep apnea (OSA) affects 5% of the adult population and its prevalence is up to 13 times higher in coronary artery disease (CAD) patients. However, OSA in this population is less symptomatic, leading to lower adherence to positive airway pressure (CPAP). While oropharyngeal exercise showed a significant decrease in apnea-hypopnea index (AHI) in patients with moderate OSA, there have been no studies testing the impact of specific inspiratory muscle training (IMT) for these patients. The aim of our study was to assess the effectiveness of IMT on AHI reduction in CAD patients with moderate OSA.

**Methods:**

We included patients with CAD involved in a cardiac rehabilitation program and presenting an AHI between 15 and 30. Patients were randomized in a 1:1 allocation to a control group (CTL – classic training) or an IMT group (classic training + IMT). IMT consisted in 60 deep inspirations a day, 6 days a week, into a resistive load device set at 70% of the maximum inspiratory pressure (MIP). After 6 weeks, we compared AHI, neck circumference, Epworth Sleepiness Scale, Pittsburgh Sleep Quality index, and quality of life with the 12-item Short Form Survey before and after rehabilitation.

**Results:**

We studied 45 patient (60 ± 9 y, BMI = 27 ± 6 kg.m^−2^). The IMT group (*n* = 22) significantly improved MIP ( *p* < 0.05) and had a significant decrease in AHI by 25% (−6.5 ± 9.5, *p* = 0.02). In the CTL group (*n* = 23), AHI decreased only by 3.5% (−0.7 ± 13.1; *p* = 0.29). Between groups, we found a significant improvement in MIP ( *p* = 0.003) and neck circumference ( *p* = 0.01) in favor of the IMT group. However, we did not find any significant improvement of AHI in the IMT group compared to CTL ( *p* = 0.09).

**Conclusion:**

A specific IMT during cardiac rehabilitation contributes to reduce significantly AHI in CAD patients with moderate OSA. Magnitude of the decrease in OSA severity could be enhanced according to implementation of specific IMT in this population.

## Introduction

Prevalence of moderate to severe obstructive sleep apnea (OSA; apnea plus hypopnea index >15 events.h^−1^) is thought to affect up to 23% of women and 49% of men (Heinzer et al., 2015). OSA is characterized by recurrent episodes of complete (apneas) or partial (hypopneas) upper airway collapse during sleep. It always stems from obstruction of the pharynx during the inspiratory phase (Fogel et al., 2005).

Several studies have shown that OSA is an independent risk marker and probably risk factor of coronary heart disease (BenAhmed et al., 2014; Tietjens et al., 2019). It is also a major risk factor for cardiovascular morbidity and mortality (Peker et al., 2000; Mooe et al., 2001; Coughlin et al., 2004; Bradley and Floras, 2009). In comparison with other OSA patients, coronary artery disease patients with OSA are characterized by relatively poor diurnal symptoms (Javaheri et al., 1998, 2017; Sin et al., 2002; Arzt et al., 2006). The absence of clinical repercussions may induce a higher withdrawal rate of standard continuous positive airway pressure (CPAP) treatment. Also, for those who have accepted CPAP, long-term adherence remains an important issue (Anandam et al., 2013; McEvoy et al., 2016; Rotenberg et al., 2016; Libman et al., 2017; Askland et al., 2020). Alternative treatments, such as mandibular advancement or surgery, can be proposed for moderate OSA or for patients with limited daytime symptoms but with limited tolerance by some patients over time (Fleury et al., 2010; Giralt-Hernando et al., 2019). Therefore, alternative treatments are needed to reach better acceptance and adherence.

Excessive relaxation of the tongue and soft palate evidenced by a decrease in the EMG pattern of the genioglossus and tensor of the palate muscles (Fogel et al., 2005; Oliven et al., 2019) partly explains the OSA pathogenesis. While physical activity has shown relative effectiveness in improving apnea-hypopnea index (AHI; Kline et al., 2011; Aiello et al., 2016; Berger et al., 2018, 2021; Hupin et al., 2018), specific exercises of the oropharyngeal area could have a more targeted impact. Thus, Guimarães et al. (2009) have shown that strengthening oropharyngeal muscles by mouth, tongue, and pronunciation exercises reduce AHI in patients with moderate OSA by 8.7 events.h^−1^. An improvement of subjective quality of sleep and a reduction in neck circumference were also found in the “strengthening oropharyngeal muscles” group. In the last decade, several other studies have investigated the effect of respiratory muscle training to decrease the impact of OSA (Vranish and Bailey, 2016; Souza et al., 2018; Ramos-Barrera et al., 2020). Indeed, the resistance strengthening of the inspiratory muscles would have a positive impact on the tone of the oropharyngeal muscles (How et al., 2007). However, these studies proposing inspiratory muscle training were carried out on small sample sizes and none of them were performed with asymptomatic coronary artery disease patients.

The objective of our study was to assess the effects of strengthening inspiratory muscles on AHI in coronary patients with moderate OSA engaged at the same time in a post-infarction cardiac rehabilitation program (CR) using a randomized controlled design. We assessed the benefit of adding an inspiratory resistance muscle training to OSA patients undergoing cardiac rehabilitation (IMT group) compared to a control group undergoing only cardiac rehabilitation (CTL group). We analyzed ventilatory polygraphy data and respiratory muscle strength before and after cardiac rehabilitation.

## Materials and Methods

### Study Design

This was a 6 week randomized controlled trial, including on the one hand a control group with cardiac rehabilitation only, and on the other hand an interventional group admitted for CR and IMT. This study was conducted in the cardiac rehabilitation department at Saint-Etienne university hospital (France). Eligible patients were randomly assigned in a 1:1 allocation.

### Patient Characteristics and Eligibility Criteria

During the initial evaluation, patients had a 24-h Holter ECG monitoring to assess cardiac arrhythmia and to evaluate their very low frequency power spectral density of heart rate increment (VLFI) which is a predictor of sleep disorder (Roche et al., 2002; Sforza et al., 2007). Patients with a high risk of OSA, attested by VLFI>4%, had a respiratory polygraphy recording to assess OSA severity more accurately. Adult patients initiating a cardiac rehabilitation, under the age of 80, with an AHI ≥15 and ≤ 30 events h^−1^ were eligible to participate.

Exclusion criteria were recent thoracic surgery by sternotomy (<12 weeks), obstructive ventilatory disorder with an FEV-1/FVC ratio <70%, already treated for OSA.

### Ethics Approval and Consent of Participants

The study was performed from May 2015 to July 2021. Approval and ethical clearance were obtained from an institutional review board (CPP Sud Est I 1408189-2015-A00030-49) which was in accordance with the principles embodied in the Declaration of Helsinki. Prior to study initiation, the objectives of the study were clearly explained to subjects in order to obtain written informed consent. The study was *a priori* registered at ClinicalTrial.gov (NCT02494648).

### Cardiac Rehabilitation CR

The CR at the Saint-Etienne University Hospital was a 7-week interdisciplinary program that combines interventions performed by cardiologists, nurses, rehabilitation physicians, physiotherapists, dieticians, and psychologists. At the time of enrollment, the patient’s functional status was assessed by a cardiopulmonary exercise test (CPET). Exercise training consists of 20 sessions, three times a week (Monday, Wednesday, and Friday) for 1.5 h. Each session starts with a 10-min warmup period followed by 25 min of cycling or treadmill (power output at ventilatory threshold defined during the initial CPET) and a 5-min cool down period. Resistance muscle training of upper and lower limbs is also offered. The workout was divided into 80% aerobic and 20% resistance exercises. The first week of CR, continuous endurance exercises were preferred, and from the second week, interval exercises were added. An expert physiotherapist and an adapted physical activity monitor supervised sessions with continuous heart rate and pulse oximetry monitoring. The workload progression was adjusted weekly according to the patient’s tolerance (Borg Perceived Effort Scale). Patients attended weekly hour-long group sessions with healthcare professionals aimed at reinforcing their health education. Particular emphasis is provided on understanding the pathophysiology of coronary artery disease, the role of cardiovascular risk factors, and its management, mainly through physical activity, anxiety control, smoking cessation, nutritional balance, and adherence to guidelines for recommended drugs.

### Interventional Group (IMT)

The interventional group followed the CR program described above, as well as a 6-week IMT. To strengthen the inspiratory muscles, we used a resistive loading device (POWERbreathe^®^ Plus medium resistance, Southam, United Kingdom) with a flow-independent one-way valve to ensure consistent resistance and an adjustable specific pressure setting (from −23 to −186 cmH_2_0). IMT consisted of performing 6 days a week, two sessions of 30 inspirations against a load of 70% MIP, during the entire duration of the CR period, i.e., nearly 40 sessions in 6 weeks. On days when the patient was present at the CR center, the IMT sessions were carried out under the supervision of the physiotherapist (PL). The third week, the MIP was reevaluated in the same conditions as during inclusion in order to adjust the training load. Patients were asked to keep a training notebook with indication of the difficulty in performing the session on a 0–5 scale.

### Control Group

Patients randomized in the control group participated only in the CR and performed the same evaluations as the IMT group at baseline and at follow-up.

### Outcome Measures

All outcome measures described were performed at baseline and at 6-week follow-up.

*Respiratory polygraphic data*. AHI was the main outcome measured from respiratory polygraphy. Ambulatory polygraphy was performed at home using either a Nox-T3 (Nox Medical, Reykjavik, Iceland) or a VistaO_2_flux (Novacor, Rueil Malmaison, France). For both devices, the signals acquired were the following: nasal flow with pressure transducer and oxygen saturation with a digital pulse oximeter (Nonin, United States). For Nox-T3, respiratory signals were recorded with chest and abdominal respiratory inductance plethysmography belts. For VistaO_2_flux, respiratory signals were evaluated by thoracic impedance sensors embedded in a multimodal ECG Holter recorder (Roche et al., 1999; Poupard et al., 2008; Chouchou et al., 2013). For each patient, the second polygraphy at the end of the study was carried out with the same device as baseline in order to be more consistent. In accordance with the 2012 American Academy of Sleep Medicine guidelines (Berry et al., 2012), an apnea was defined as a cessation in airflow (≥90% baseline) for ≥10 s, and a hypopnea was defined as a reduction in airflow (≥30% baseline) for ≥10 s which resulted in a ≥ 4% oxygen desaturation. Sleep apnea severity was rated in accordance with the individual’s AHI, defined as the total number of apneas and hypopneas per hour of sleep and oxygen desaturation per hour of sleep; where an AHI of 5–15 events.h^−1^ was defined as mild, 16–30 moderate, and >30 designated as severe OSA. Secondary polygraphic outcomes included oxygen desaturation index (ODI), central apnea index, the mean and the lowest oxyhemoglobin saturation (SpO_2_), and the percentage of time with a SpO_2_ under 90% (SpO2 < 90%).

*Respiratory function assessment*. Lung function was assessed by standard spirometry and included forced expiratory volume in 1 s (FEV-1), forced vital capacity (FVC), and peak expiratory flow in accordance with the guidelines of the European Respiratory Society (Laveneziana et al., 2019). Maximal inspiratory pressure (MIP) was obtained with a specific device (POWERbreathe KH2, POWERbreathe International Ltd., Southam, United Kingdom). The maneuvers were carried out with the patient seated and knees flexed to 90° and were performed using the residual volume to realize a forced inspiration. Maximal inspiratory pressure was determined as the highest average pressure over 1 s reached during exercise. We considered three valid maneuvers (coefficient of variation below 10%) and the highest value was used for IMT.

*Surveys*. We analyzed questionnaires evaluating subjective sleep quality and quality of life. Subjective daytime sleepiness was measured by the Epworth Sleepiness Scale (ESS) which evaluates the propensity to sleep from “no” (scored 0) to “intense” (scored 3) in eight different situations. A total score > 10 indicates excessive daytime sleepiness (Johns, 1991). Quality of sleep was evaluated with the Pittsburgh Sleep Quality Index (PSQI), which is a questionnaire that evaluates seven sleep components on a scale from 0 (no difficulty) to 3 (severe difficulty). The results are expressed as a global score (ranging from 0 to 21). A total score > 5 indicates poor sleep quality (Buysse et al., 1989). The 12-item Short Form Survey (SF-12) was used to assess self-reported health-related quality of life, which evaluates physical health with a range of 23.99 (poor) to 56.58 (good) and mental health with a range of 19.06 (poor) to 60.76 (good; Ware et al., 1996).

### Statistical Data Analysis

Descriptive and inferential analyses were performed using GraphPad Prism 6 (GraphPad, San Diego, United States). Characteristics of the sample and treatment effect between groups were assessed using Student’s *t*-test for independent samples. Pre-versus post-CR differences were examined using paired *t*-tests, and chi-square test corrected by Fisher’s exact test was used for the categorical variables. The results are shown as mean ± standard deviation, number and percentage, and as differences of means and confidence interval (95%). Statistical significance was set at a two-tailed *p* < 0.05.

The sample size calculation was based on the Puhan et al. (2006) study results. For an expected difference of 7 points of AHI between the two groups, with a statistical power of 90%, we calculated a sample size of 44 patients. To consider dropouts before the end of the rehabilitation program, we decided to include 48 patients.

## Results

### Demographics and Baseline Characteristics

**Figure 1:**
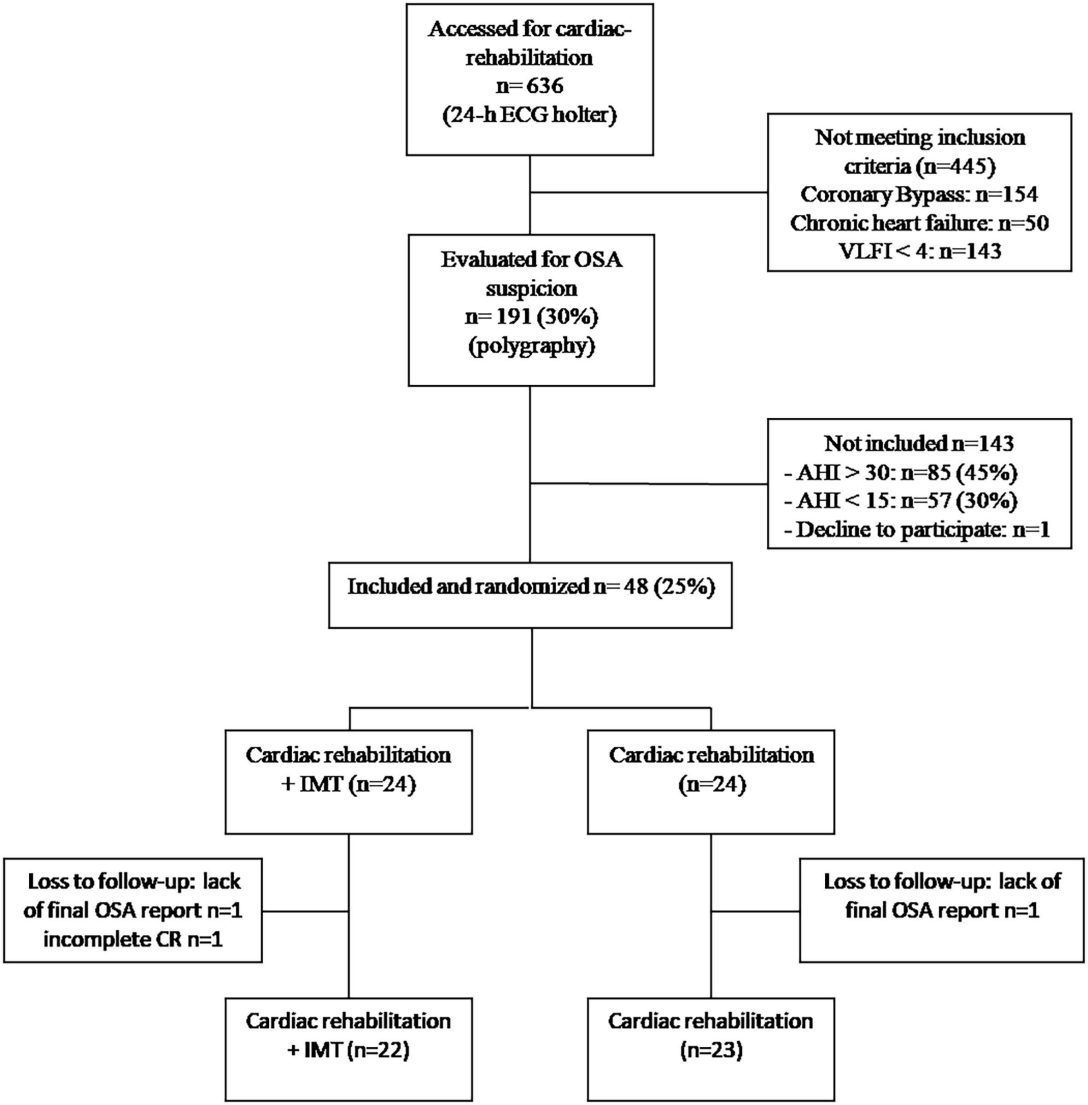
Flowchartof the study. OSA, obstructive sleep apnea; AHI, apnea-hypopnea index; IMT, inspiratory muscle training; CR, cardiac rehabilitation; and VLFI, very low frequency power spectral density of heart rate increment.

During the inclusion period, 636 patients were admitted to our cardiac rehabilitation center and 191 patients (40%) were assessed for suspected OSA. After respiratory polygraphy, 44.5% (*n* = 85) had AHI > 30 events.h^−1^ and 29.8% (*n* = 57) had AHI < 15events.h^−1^, and 48 patients with an AHI between 15 and 30 events.h^−1^ were included in our study. The flowchart of the study is depicted in Figure 1. Baseline clinical characteristics of the study population are summarized in Table 1. Analyses of characteristics showed no significant differences at baseline.

**Table 1:** Baseline characteristics.

	CTL Group *n* = 23	IMT Group *n* = 22	Value of *p*
Age (y)	59.3 ± 10.3	61.0 ± 8.4	0.55
Females	4 (18)	1 (4)	
Height (cm)	173.0 ± 8.4	169.5 ±	0.21
Weight (kg)	83.9 ± 18.9	81.0 ±14.1	0.58
BMI	28.1 ± 5.8	27.9 ±4.1	0.91
Neck size (cm)	40.1 ± 2.6	38.8 ±2.8	0.15
High blood pressure	8 (35)	10 (42)	0.64
Dyslipedemia	9 (39)	7 (29)	0.55
Smokers	13 (57)	14 (58)	0.65
Diabetes	6 (26)	7 (29)	0.82
Obesity	6 (26)	9 (38)	0.41
LVEF (%)	53.9 ± 9.6	57±9.4	0.28
Coronary intervention			
Thrombolysis	3 (13)	6 (25)	0.31
Angioplasty without stent	3 (13)	3 (13)	0.96
Single-stem stent	13 (57)	10 (42)	0.25
Multi-stem stents	8 (34)	10 (42)	0.53
Treatment			
Beta blocker	20 (87)	18 (75)	0.31
Aspirin	21 (91)	21 (88)	0.68
Double APT	23 (100)	23 (96)	0.33
Statin	21 (91)	17 (71)	0.08
ACEI/ARB	19 (83)	18 (75)	0.53
FEV-1%	99.0 ± 13.7	97.2±19.1	0.78

*Values are mean ± standard deviation or n (%). BMI, body mass index; LVEF, left ventricular ejection fraction; APT, anti-platelet; ACEI/ARB, angiotensin-converting enzyme inhibitor/angiotensin receptor blocker; and FEV-1, Forced expiratory volume in 1 s*.

### Change in Characteristics

The post-intervention change in BMI was not significantly different between groups (Table 2). In the IMT group, neck circumference decreased by 0.5 ± 0.9 cm (*p* = 0.02), and the difference between groups was also significant after intervention (*p* = 0.01). MIP significantly increased with the IMT intervention compared to the control (*p* = 0.003; Table 2).

**Table 2:** Average values of characteristics and cardiopulmonary exercise test obtained before and after cardiac rehabilitation in the control (CTL) and inspiratory muscle training (IMT) groups.

	CTL group			IMT group			Value of *p* intergroup
	Before	After	Value of *p*	Before	After	Value of p	
Characteristics							
Weight (kg)	81.0 ± 14.1	83.9 ± 18.9	0.92	83.9 ± 18.9	80.2 ± 14.3	0.09	0.16
BMI	27.9 ± 4.1	28.1 ± 5.9	0.83	28.1 ± 5.8	27.4 ± 3.8	0.07	0.11
Neck circumference (cm)	40.1 ± 2.6	40.5 ± 2.2	0.18	38.8 ± 2.8	38.3 ± 3.1	**0.02**	**0.01**
MIP (cmH_2_O)	89.6 ± 17.7	92.4 ± 22.3	0.65	97.6 ± 29.7	115.4 ± 30.2	**0.0004**	**0.003**
Cardiopulmonaryexercise test							
VO_2_ peak (ml^−^1.min^−^1.kg^-1^)	20.6 ± 4.9	23.7 ± 5.3	**0.0003**	20.7 ± 4.9	24.7 ± 5.6	**<0.0001**	0.19
Pmax (W)	133.6 ± 39.3	164.4 ± 39.2	**<0.0001**	136.9 ± 32.3	159.4 ± 48.9	**<0.0001**	0.59
Heart rate at peak (% predicted)	75.3 ± 11.9	81.9 ± 13.5	**0.02**	79.8 ± 15.9	80.7 ± 14.1	0.57	0.15

*Values are mean ± standard deviation. Significant values (p < 0.05) are indicated in bold. BMI: body mass index; MIP: maximum inspiratory pressure; VO2peak: maximal oxygen uptake; Pmax: maximal power output.*.

### Cardiopulmonary Exercise Test

At the end of the cardiac rehabilitation, both groups showed a significant improvement in maximal oxygen uptake from 20.7 ± 4.9 before CR to 24.7 ± 5.6 (ml^−1^min^−1^kg^−1^; *p* < 0.0001) after CR for the IMT group and from 20.6 ± 4.9 before CR to 23.7 ± 5.3 (ml^−1^min^−1^kg^−1^; *p* = 0.0003) after CR for the control group. Maximum power output during CPET after CR increased by 23.6 ± 23.7 W (*p* < 0.0001) for the IMT group and by 27.5 ± 22.9 W (*p* < 0.0001) for the control group Table 2.

### Polygraphic Data

**Figure 2:**
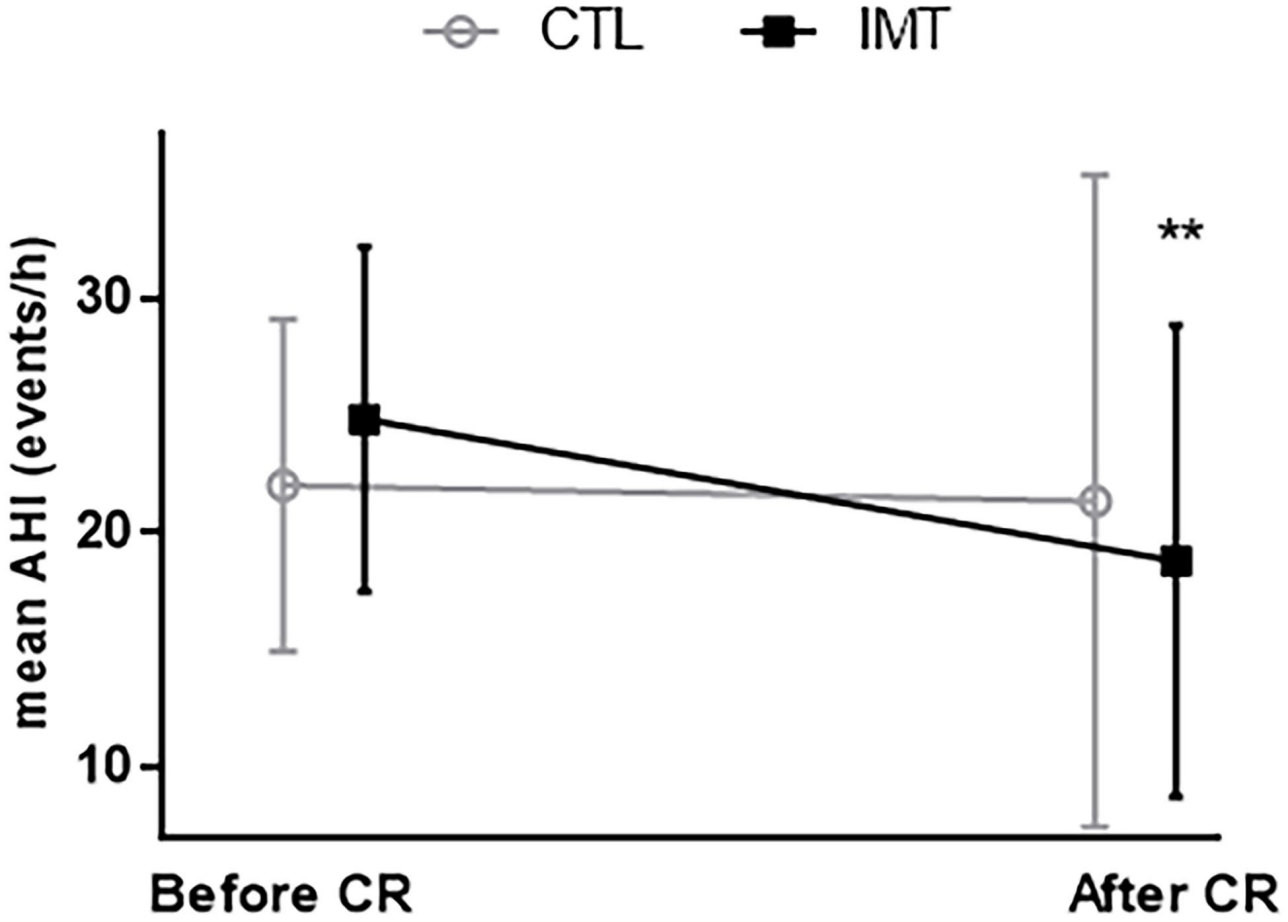
Evolution of apnea-hypopnea index (AHI) before and after cardiac rehabilitation (CR). ^**^p = 0.003; significant decrease of AHI after cardiac rehabilitation only for IMT group (black square).

At baseline, polygraphy data between groups were similar (Table 3). Post-intervention, the mean reduction in AHI score was only significant for the IMT group, which showed a decrease of 6.5 ± 9.5 events.h^−1^ (95% CI: 2.015 to 10.08, *p* = 0.005). By contrast, the control group which only carried out the CR showed a nonsignificant decrease of 0.7 ± 13.4 events.h^−1^ (Table 3; Figure 2). The difference in AHI of 5.8 ± 3.2 points between the two groups was close to significance (*p* = 0.09). ODI was significantly decreased in the IMT group by 6.4 ± 8.02 events.h^−1^ (95% CI: −9.910 to −2.977, *p* = 0.0009); for the CTL group, no significant change in ODI was observed (*p* = 0.14). If we only consider the obstructive part of sleep apnea (AHI minus central apnea index), we observed a significant decrease of 6.7 ± 8.5 events.h^−1^ (*p* = 0.004).

**Table 3:** Average values of polygraphy and questionnaires before and after cardiac rehabilitation in the control (CTL) and inspiratory muscle training (IMT) groups.

	CTL group			IMT group			
	Before	After	Value of *p*	Before	After	Value of *p*	Value of *p* intergroup
Polygraphic recording							
AHI (events.h^−1^)	22.0 ± 7.1	21.4 ± 13.9	0.29	24.9 ± 7.8	18.8 ± 10.1	**0.003**	0.09
ODI (events.h^−1^)	22.5 ± 5.2	21.4 ± 14.1	0.14	23.1 ± 8.0	16.6 ± 9.6	**0.0004**	0.12
Hypopnea Index (events.h^−1^)	14.1 ± 5.1	14.8 ± 13.1	0.45	15.4 ± 4.9	11.8 ± 6.1	**0.03**	0.15
Obstructive apnea Index (events.h^−1^)	6.9 ± 3.5	6.4 ± 4.6	0.33	6.3 ± 5.6	4.0 ± 3.2	**0.02**	0.29
Obstructive part of OSA (events.h^−1^)	21.2 ± 4.9	21.8 ± 16.0	0.29	21.8 ± 7.1	15.8 ± 7.7	**0.004**	0.09
Central apnea Index (events.h^−1^)	1.7 ± 2.3	1.1 ± 1.1	0.37	2.4 ± 2.9	1.4 ± 1.8	0.32	0.78
Lowest SpO_2_ (%)	83.8 ± 2.7	82.9 ± 5.2	0.57	83.3 ± 4.1	85.7 ± 2.2	0.08	0.12
Mean SpO_2_ (%)	92.5 ± 1.6	92.1 ± 1.7	0.52	93.0 ± 1.4	93.4 ± 1.4	0.30	0.14
SpO2 < 90% (min)	13.7 ± 17.5	24.8 ± 57.5	0.33	12.3 ± 26.6	4.6 ± 6.3	0.25	0.13
Questionnaires							
ESS	7.4 ± 3.6	6.2 ± 4.0	0.24	7.3 ± 5.2	4.3 ± 2.9	**0.02**	0.26
PSQI	6.0 ± 3.7	5.4 ± 3.2	0.28	6.1 ± 3.7	3.9 ± 1.6	0.053	0.38
SF-12 mental score	46.4 ± 9.6	48.8 ± 8.8	0.68	44.6 ± 11.2	52.3 ± 9.6	**0.008**	**0.013**
SF-12 physical score	44.4 ± 9.6	44.9 ± 10.5	0.80	41.2 ± 10.0	42.6 ± 12.5	0.30	0.70

*Values are mean  ±  standard deviation. AHI, apnea-hypopnea index; ODI, oxygen desaturation index, OSA, obstructive sleep apnea; SpO_2_, oxygen saturation value; ESS, Epworth Sleepness Scale; PSQI, Pittsburgh Sleep Quality Index; and SF-12, 12-itemShort Form Survey. Significant values (p < 0.05) have been indicated in bold*.

### Questionnaires

ESS only decreased significantly for the IMT group (−2.2 ± 3.2 points, *p* = 0.0176), and PSQI was close to significant with a mean score below 5 (3.9 ± 1.6, *p* = 0.053). For the mental health score of SF-12, the IMT group showed a significant increase of 5.58 ± 7.4 points (*p* = 0.008), while it did not change in the CTL group (*p* = 0.7). As a result, we found for this mental health score a significant difference between groups of 6.5 ± 2.6 points (*p* = 0.01). We found no other significant difference in the questionnaires, either intragroup or intergroup (Table 3).

## Discussion

Our study showed that 6 weeks of resistance IMT in patients with moderate OSA admitted in cardiac rehabilitation for coronary artery disease allowed a decrease in AHI and ODI. This is in line with the effect of IMT on pharyngeal tone. In this sense, How et al. (2007) showed in awake subjects with calm breathing that an acute bout of inspiratory muscle exercise allowed to increase the activation of specific upper airway dilator muscles and that an IMT could, by increasing the passive tone of the upper airway dilatators, be more favorable in OSA patients with greater compliance of their pharyngeal wall (Isono et al., 1997).

In our study, patients undergoing IMT showed an improvement of their MIP (+ 18%). Although not measured, we hypothesize that IMT, initially focused on improving diaphragmatic strength and probably endurance, also allowed an improvement of the upper airway dilatator muscle tone during sleep. The decrease in the obstructive part of the sleep disorders recorded (AHI minus central apnea index) suggests that IMT could reduce collapsibility of the upper airway during sleep, even in the absence of neural drive. According to a study by Guimarães et al. (2009), in patients who performed oropharyngeal exercises for 12 weeks, the authors suggested an upper airway remodeling linked to a significant decrease in neck circumference. They also showed that these changes in neck circumference were negatively correlated with improvement in AHI. Finally, in our study, the significant decrease in neck circumference might be related to the improvement in the tone of the upper airway, which would require further investigational studies.

Noteworthy, IMT allowed a significant reduction in hypoxemic load in the present study. In fact, in the IMT group, the patients presented a significantly lower ODI and a decrease in the time spent below 90% of SpO2 after intervention. The improvement of these factors is of strong interest in the management of coronary patients with OSA considering the major role of intermittent hypoxemia in terms of morbidity and mortality as well as in the development of cardiovascular risk factors (Garvey et al., 2009; Dewan et al., 2015).

Our results suggest that, at baseline, coronary patients with moderate OSA present few OSA-related symptoms. Indeed, the subjective daytime sleepiness scores assessed by the Epworth questionnaire before CR were <10 (which would indicate excessive daytime sleepiness) in the whole population, while the patients reported poor sleep quality. Of note, cardiac rehabilitation had no significant effect on these two scores in the control group. On the other hand, in the IMT group, the significant improvement in AHI is in line with a significant improvement in daytime sleepiness as well as an improvement of sleep quality. Due to the high prevalence of OSA in the population with coronary artery disease (69% of our patients were assessed for suspicion of OSA; Hupin et al., 2018), it seems important to systematically look for sleep disorders in patients who have had a heart attack.

## Strengths and Limitations

The strength of our study was its randomized controlled design with an easy access IMT device, an IMT simple to perform and requiring little daily training time (60 repetitions per day, i.e.,20 min). Patient feedbacks were very positive, whereby most found a benefit and continued IMT after the end of their cardiac rehabilitation. Another strength of our study is the centralized blind reading of the polygraphic signals avoiding any analysis bias.

Our study also had limitations. First, we did not find any significant difference between the group performing IMT in addition to CR compared to the group performing CR alone. This lack of difference could be explained by the fact that physical training *per se*, *as* provided during CR, could improve the AHI in some patients included in the control group (Berger et al., 2018). Indeed, physical exercise could decrease fat mass (Kline et al., 2011; Desplan et al., 2014), greater overall muscle tone, and improved respiratory function (Sengul et al., 2009). This set of elements may have reduced the impact of IMT in our study. Indeed, physical training induces an increase in voluntary ventilation which, in the long term, allows better ventilatory control, an increased sensitivity to respiratory drive, and an increase in the strength of the inspiratory muscles (a median increase of +7 cmH_2_O was observed for the control group and a maximum gain of 22 cmH_2_O for a patient). This slight increase in MIP may have resulted in a lower rate of complete obstructive apnea in favor of a higher rate of hypopnea in the control group.

Another limitation to note is the lack of objective evaluation of adherence to IMT training since the device used is mechanical and does not have a repetition counter. For the sake of more precise evaluation, the use of an electronic device would have allowed rigorous monitoring of the training. Finally, our study did not allow to establish a causality link between improved MIP and increased resistance to upper airway collapse during sleep. Future physiological studies evaluating the effect of IMT on upper airway closure pressures during sleep are needed to confirm our data.

## Clinical Implication and Conclusion

In case of CPAP treatment failure, it appears necessary to offer various alternatives to OSA patients to reduce the severity of sleep disorders. In that view, our study brings a new piece to the puzzle by showing that a simple IMT exercise, performed daily, could reduce OSA severity in nontreated patients. In the most severe patients, this treatment could also be offered in addition to CPAP to reduce the pressures insufflated into the airways and thus improve patient comfort. Further investigational studies are needed to determine the best IMT modality (strength training or endurance training) and the potential combined effect with expiratory muscle training, as previously shown by Puhan et al. (2006) and Ward et al. (2012).

In conclusion, IMT for 6 week decreases AHI in coronary patients with moderate asymptomatic OSA. IMT could be an adjuvant therapy for patients who are unwilling or unable to tolerate nightly CPAP.

## Data Availability Statement

The raw data supporting the conclusions of this article will be made available by the authors, without undue reservation.

## Ethics Statement

The studies involving human participants were reviewed and approved by Saint-Etienne University Hospital ethics committee: CPP Sud Est I 1408189-2015-A00030-49. The patients/participants provided their written informed consent to participate in this study.

## Author Contributions

PL, MB, and DH: writing of original draft. All authors had a substantial contribution to the conception and design of the study. PL, MB, AZ, FR, and DH: responsible for data collection and formal analysis. MB, J-CB, FR, DH, and AG: supervision. All authors contributed to the article and approved the submitted version.

## Conflict of Interest

The authors declare that the research was conducted in the absence of any commercial or financial relationships that could be construed as a potential conflict of interest.

## Publisher’s Note

All claims expressed in this article are solely those of the authors and do not necessarily represent those of their affiliated organizations, or those of the publisher, the editors and the reviewers. Any product that may be evaluated in this article, or claim that may be made by its manufacturer, is not guaranteed or endorsed by the publisher.
